# Cigarette Smoke-Induced Respiratory Response: Insights into Cellular Processes and Biomarkers

**DOI:** 10.3390/antiox12061210

**Published:** 2023-06-03

**Authors:** Sang-Ryul Cha, Jimin Jang, Sung-Min Park, Se Min Ryu, Seong-Joon Cho, Se-Ran Yang

**Affiliations:** Department of Thoracic and Cardiovascular Surgery, School of Medicine, Kangwon National University, 1 Kangwondaehak-gil, Chuncheon 24341, Republic of Korea

**Keywords:** cigarette smoke, oxidative stress, COPD, NF-κB, MAPK, RAGE, Nrf2, PF, cancer, ARDS, AE-COPD, CVD

## Abstract

Cigarette smoke (CS) poses a significant risk factor for respiratory, vascular, and organ diseases owing to its high content of harmful chemicals and reactive oxygen species (ROS). These substances are known to induce oxidative stress, inflammation, apoptosis, and senescence due to their exposure to environmental pollutants and the presence of oxidative enzymes. The lung is particularly susceptible to oxidative stress. Persistent oxidative stress caused by chronic exposure to CS can lead to respiratory diseases such as chronic obstructive pulmonary disease (COPD), pulmonary fibrosis (PF), and lung cancer. Avoiding exposure to environmental pollutants, like cigarette smoke and air pollution, can help mitigate oxidative stress. A comprehensive understanding of oxidative stress and its impact on the lungs requires future research. This includes identifying strategies for preventing and treating lung diseases as well as investigating the underlying mechanisms behind oxidative stress. Thus, this review aims to investigate the cellular processes induced by CS, specifically inflammation, apoptosis, senescence, and their associated biomarkers. Furthermore, this review will delve into the alveolar response provoked by CS, emphasizing the roles of potential therapeutic target markers and strategies in inflammation and oxidative stress.

## 1. Introduction

The World Health Organization (WHO) has estimated that smoking causes about 8 million deaths each year, with the majority occurring in low- and middle-income countries [1]. Despite estimates, more than 100 million people around the world continue to smoke regularly [2]. To combat this issue, various world health institutions have set agreements and goals to reduce the prevalence of smoking. Representatively, the WHO Framework Convention on Tobacco Control (FCTC) has come into effect since 2005, and 181 countries are participating as of 2020 [3]. The FCTC has implemented measures such as tobacco price and tax increases, packaging and labeling requirements, and health warning labels [4,5]. Due to these policies, the global average number of smokers is decreasing; however, smoking remains a significant cause of disease and death for individuals.

Cigarette smoke (CS) is a representative risk factor for vascular and various organ diseases, as well as respiratory illness [6]. With over 4000 chemicals, such as nicotine, acrolein, phenols, carbon monoxide, benzene, and formaldehyde [7,8]. Both active and passive inhalation of these harmful chemicals can induce oxidative stress in the body, leading to inflammation, apoptosis, and senescence. Acrolein, for example, causes mutations by forming acrolein-induced DNA adducts [9]. Acrylonitrile is a representative oxidant in cigarettes; it produces 8-oxo-2′-deoxyguanosine and contributes to oxidative stress [10].

CS contains a high level of reactive oxygen species (ROS), which can overwhelm the body’s antioxidant defense system and result in oxidative stress [11]. Chronic CS exposure causes persistent oxidative stress in the lung, damaging the respiratory system and increasing the risk of various diseases. The detrimental effects of smoking on the lung can be attributed to both the direct and indirect consequences of oxidative stress [12]. Direct effects include damage to cellular components such as lipids, proteins, and DNA [13]. Indirect effects involve the activation of inflammatory cells, leading to the release of pro-inflammatory cytokines and chemokines that further exacerbate oxidative stress and cause tissue damage [14].

The production of ROS is involved in various cells, and mitochondria and NADPH oxidase (NOX) are the main sources of ROS [15]. During ATP synthesis by oxidative phosphorylation, the main function of mitochondria, ROS is mainly produced, and the generated ROS is removed by superoxide dismutase, peroxiredoxin, and glutathione peroxidase [16,17]. NADPH oxidase is found in neutrophils and vascular cells and is involved in the generation of neutrophil extracellular traps (NETs) and vasoconstriction and vasodilation, depending on the cell [18,19].

Oxidative stress can inflict cellular damage through the oxidation of lipids, proteins, and DNA [20]. For example, lipid peroxidation leads to the formation of aldehydes and other toxic compounds that can disrupt cellular membranes [21]. The oxidative modification of lipids can lead to membrane permeability changes and cellular dysfunction, ultimately leading to cell death [22]. Protein oxidation may result in structural changes and loss of function, while DNA oxidation can lead to mutations and cell death [23]. In addition, this stress has been linked to a number of lung diseases, including chronic obstructive pulmonary disease (COPD) and lung cancer [24]. Oxidative stress induces inflammation and damage, leading to several respiratory diseases such as COPD, ARDS, lung cancer, and pulmonary fibrosis, as well as cardiovascular diseases [25]. Further research is necessary to comprehensively understand how oxidative stress affects the lung and to identify effective antioxidants for the treatment of lung diseases [26,27,28]. In addition, investigating the role of oxidative stress in the development of lung diseases and determining the most effective strategies for prevention and treatment are also crucial [29]. In this review, we aim to provide a comprehensive summary of the impacts of oxidative stress, primarily triggered by cigarette smoke, on cellular processes such as inflammation, apoptosis, aging, and autophagy. We will further explore the consequential diseases and potential therapeutic targets in the lungs and cardiovascular system.

## 2. Cellular Responses to Cigarette Smoke

### 2.1. Inflammation

Inflammation is a biological defense mechanism against harmful stimuli such as pathogens and damaged cells [30]. It can be acute or chronic, with CS often being a risk factor for chronic inflammation [31]. Smoking induces high concentrations of cigarette smoke particles to enter the lungs, and these stimuli are harmful factors that cause acute or chronic inflammation [32,33,34,35]. Oxidative stress is the primary cause of inflammation resulting from smoking [36]. During oxidative stress, cell damage leads to the release of damage-associated molecular patterns (DAMP) molecules [37]. The receptor for advanced glycation end products (RAGE) is a 35-kilodalton membrane receptor known as a receptor for DAMP molecules such as AGE, high mobility group box 1 (HMGB1), and S100 families [38]. RAGE, also known as a marker for type 1 lung epithelium, is closely related to lung disease [39]. RAGE has a soluble form (sRAGE) secreted out of the cell and a membrane-bound form (mRAGE). In general, the soluble form binds to DAMP instead of membrane-bound RAGE to act as an antagonist to inhibit the activation of membrane-bound RAGE due to cigarette smoke [40,41,42,43]. Toll-like receptors (TLRs) are also key receptors for DAMP and play a crucial role in the innate immune response [44]. TLRs are commonly found in various innate immune cells, such as macrophages and dendritic cells, and recognize pathogen-derived molecules [45]. TLRs are significant mediators in the inflammatory mechanism caused by smoking. Barua et al. reported that CS triggers inflammation through the activation of the histamine receptor-1 (H1R)-TLR2/4-cyclooxygenase2 (COX2) axis [46]. Moreover, Nadigel et al. confirmed the expression and inflammation of high TLR4/9 in COPD patients [47]. As such, oxidative stress and DAMP-induced inflammation by CS are essential mechanisms in the immune response. 

And activation of DAMP receptors, such as RAGE and TLRs, typically activates the mitogen-activated protein kinase (MAPK) and nuclear factor kappa-light chain-enhancer of activated B cells (NF-κB) pathways [48]. NF-κB is a p50/p65 heterodimer that plays an important role in inflammation, immune response, and apoptosis [49,50]. IκB present in the cytoplasm under physiological conditions inhibits NF-κB, and phosphorylated IκB through activation of IκB kinase (IKK) promotes phosphorylation and translocation of NF-κB to induce it to act as a transcription factor [51]. These activated NF-κBs regulate the recruitment of various infectious cytokines and inflammatory cells [52]. MAPK is a family of protein kinases that regulate cellular responses to various stimuli, such as thermal shock, osmotic stress, and pro-inflammatory cytokines [53]. MAPK typically includes extracellular signal-regulated kinases (ERK1/2), p38, and c-Jun N-terminal Kinase (JNK1/2). Among them, ERK is activated by differentiation signals, and JNK and p38 are activated by inflammation and various stresses [53,54]. Each activated factor regulates the expression of pro-inflammatory cytokines. The secretion of inflammatory cytokines by activation of these factors activates the signal transducer and activator of transcription (STAT) pathway [55,56]. The phosphorylated STAT controls the cell cycle, apoptosis, and differentiation along with interferon regulatory factor 9 (IRF9), CREB-binding protein (CBP)/P300 [57,58,59].

Various cells are involved in immune responses, but macrophages, neutrophils, and T cells play a major role in innate and adaptive immunity. Macrophages are crucial components of the innate immune system that recruit and activate lymphocytes through phagocytosis and antigen presentation [60]. They can be polarized into M1 (pro-inflammatory) and M2 (anti-inflammatory) cells [61]. The relationship between CS and macrophage polarization has produced conflicting results. Generally, studies have shown that cigarette smoking upregulates the secretion of IL-8 in human macrophages [62,63] and releases pro-inflammatory cytokines such as IL-8 and TNF-α in rat lungs [64]. However, it has been reported that CS extract (CSE) inhibits the phagocytic response in COPD-derived alveolar macrophages [65] and that exposure to CS promotes M2 polarization in mouse macrophages [66]. Macrophage M1/M2 polarization progressively increases with the severity of smoking and disease [67]. Therefore, smoking affects the polarization of macrophages, but the direction of polarization varies depending on species and severity. Recently, Keshav et al. combined plant extracts of berberine and liquid crystalline nanoparticles to confirm antioxidant action in RAW 264.7 cells exposed to CSE [68]. As such, future research is needed to understand the impact of smoking on macrophage function. 

Neutrophils are the most abundant granulocytes and play a pivotal role in innate immunity [69]. They are essential in the pathogenesis of COPD, as emerging studies support the hypothesis that neutrophil elastase breaks down alveolar elastin, leading to emphysema [70,71,72,73]. Guzik et al. reported that acute exposure to CS in vitro induces neutrophil necrosis, promoting phagocytosis in macrophages [74]. On the other hand, Noda et al. found that CS impaired alveolar macrophage phagocytosis of apoptotic neutrophils in COPD patients [75]. Furthermore, CS-induced COPD mouse models exhibited that necroptosis and DAMP release cause airway inflammation by neutrophils [76]. In other words, direct CS exposure to neutrophils induces apoptosis; however, the resulting DAMPs can be delivered as an inflammatory inducer for other neutrophils or macrophages. Therefore, inhibition of neutrophil activity in inflammation could be an effective therapeutic target.

T cells are a major subgroup of immune cells that mediate adaptive immunity. These T cells differentiate into helper, effector, memory, and regulatory T cells upon activation [77,78,79,80]. These T cells serve different functions depending on the specific antigen. Helper T cells’ inflammatory responses can be largely classified into Th1, Th2, and Th17 [81]. Th17 cells are predominantly regulated by CS. In COPD mice, CS exposure has been shown to upregulate Th17-related cytokines, such as IL-6, IL-17A, and IL-23 [82]. Additionally, Th1 and Th17 cells significantly increased in the bronchoalveolar lavage (BAL) fluid of mice exposed to CS for 6 months [83]. Th2 cells are associated with CS-induced airway inflammation. Hove et al. confirmed increased Th2 activity and expression of IL-13 in the airways of mice exposed to CS for 4–8 weeks [84]. It has also been reported that the expression of thymic stromal lymphopoietin, which is essential for Th2 activity, is increased in the nasal cavities of mice [85]. In summary, Th1 and Th17 develop an inflammatory response with several pro-inflammatory cytokines in lung diseases caused by CS, and in the nasal cavity, a Th2-induced allergic reaction can be caused by CS.

CD8^+^ T cells play a crucial role in the host immune response by eliminating infected or damaged cells. Typically, it has been reported that inflammation and emphysema caused by CS were suppressed in CD8 knockout mice [86]. CS also activates CD8^+^ T cells in COPD patients and increases expression of IL-1β, IL-6, IL-10, IL-12, TNF-α, and IFN-γ [47]. In addition, the activation of CD8^+^ T cells by smoking has been reported in autoimmune diseases such as rheumatoid arthritis [87]. In conclusion, most studies have shown that smoking increases the activation of CD8^+^ T cells. Therefore, inhibition of CD8^+^ T cells may be an effective therapeutic target for inhibiting cellular damage caused by smoking. 

Most researchers are conducting inflammatory studies with immune cells as targets. However, there may be various mechanisms related to CS-induced inflammation. For example, CS-induced inflammation is also associated with the microbiome. Lingyue et al. reported that fermented black barley enriches probiotics such as Oscillospira and Ruminococcus, which can inhibit CS-induced lung inflammation [88]. These microbiome studies can be a new topic for the study of CS-induced inflammation. Therefore, many studies should be conducted on various targets for CS-induced inflammation.

### 2.2. Cigarette Smoke-Induced Cell Death

Apoptosis, or programmed cell death, is a highly regulated biological process crucial for maintaining tissue homeostasis and preventing disease development [89]. It features morphological changes, such as cell shrinkage, chromatin condensation, and apoptotic body formation, and is initiated by signals like cellular stress (e.g., oxidative stress) and extracellular signals (e.g., cytokines and growth factors) [90]. Apoptosis regulation is complex, involving multiple signaling pathways and target genes, including the B-cell lymphoma 2 (BCL-2) family of proteins, caspases, the tumor protein p53 (TP53), and inhibitors of apoptosis proteins (IAPs) [91]. In terms of the signaling pathways involved in apoptosis, there are two main pathways: the intrinsic pathway, initiated by signals, and the extrinsic pathway, initiated by extracellular signals [92]. The intrinsic pathway is regulated by the BCL-2 family of proteins and the activation of caspases, while the extrinsic pathway is regulated by signaling molecules such as TNF and Fas ligand [93]. The BCL-2 family of proteins plays a central role in regulating the intrinsic apoptotic pathway [94]. Pro-apoptotic members, including BAK and BAX, are responsible for releasing pro-apoptotic factors from the mitochondria in response to cellular stress, while anti-apoptotic members, such as BCL-2 and BCL-XL, inhibit apoptosis induction [95]. The activation of caspases, a family of proteases central to the execution of apoptosis, is another key step in the induction of apoptosis [96]. TP53 and the IAPs also play significant roles in apoptosis regulation, with TP53 regulating the cell cycle and the IAPs acting as inhibitors of caspase activity [97,98]. 

Caspase activation has been shown to increase in smokers, indicating an upregulation of apoptotic signaling pathways [99]. Cigarette smoke has been associated with various molecular and cellular changes in the lung tissue, including the upregulation of testis-specific serine/threonine kinase 4 (TSSK4) in alveolar epithelial type-II cells, the involvement of the SENP1-SIRT1 pathway in hyperoxia-induced alveolar epithelial cell injury, and the effects on the mouse alveolar epithelial cell line MLE 12. The upregulation of TSSK4 in alveolar epithelial type-II cells increases susceptibility to cigarette smoke-induced lung injury, as smoke exposure leads to oxidative stress, TSSK4 activation, and subsequent apoptosis of these cells, which collectively contribute to the development of lung disease-like emphysema [100]. Cigarette smoke exposure exacerbates hyperoxia-induced alveolar epithelial cell injury by dysregulating the SENP1-SIRT1 pathway, impairing the cells’ ability to cope with oxidative stress, and ultimately leading to increased cell death and lung tissue damage [101]. The dysregulation of long noncoding RNA uc.375 has been implicated in epithelial cell apoptosis and smoking-related lung diseases, including bronchopulmonary dysplasia [102]. In COPD, increased apoptosis levels in lung cells lead to the destruction of lung tissue and emphysema development [103]. In lung cancer, dysregulated apoptosis allows cancer cells to avoid programmed cell death and continue dividing and growing uncontrollably [104]. 

Apoptosis, along with necrosis and ferroptosis, three distinct forms of cell death, play crucial roles in lung injury and disease [105]. Necrosis, primarily triggered by injury or infection, leads to irreversible cellular damage [106]. This process can be mitigated by substances like ginkgolide C, which acts on the CD40/NF-κB pathway, is a significant mediator of necrosis-induced epithelial cell damage, and is considered a potential driver in COVID-19-induced acute respiratory distress syndrome [107]. On the other hand, ferroptosis is a regulated form of cell death involving iron-dependent lipid peroxides, closely linked to diseases instigated by cigarette smoke exposure [108]. Notably, several genes, such as NAD(P)H dehydrogenase [quinone] 1 (NQO1), aldo-keto reductase family 1 member C3 (AKR1C3), and glutathione peroxidase 2 (GPX2), identified through gene expression dataset analysis, have been implicated in ferroptosis and exhibit promising diagnostic potential [109].

A study showed that CS extract increased oxidative stress and apoptosis in lung cells [110]. In particular, smoking has been shown to increase the levels of pro-apoptotic factors such as BAX and decrease the levels of anti-apoptotic factors such as BCL-2 in lung cells [111]. Another study found that CS extract caused mitochondrial dysfunction and the release of pro-apoptotic factors, leading to apoptosis in lung cells [112]. As mentioned, CS also activates the extrinsic apoptotic pathway through the upregulation of death receptor signaling molecules such as Fas and TNF [113]. In lung cancer, TP53 tumor suppressor gene mutations, which regulate apoptosis and cell cycle arrest, are common and can lead to dysregulation of apoptosis [114]. Lung cancer apoptosis dysregulation may also involve alterations in the expression of BCL-2 family proteins, such as the downregulation of the pro-apoptotic protein BAX and the upregulation of anti-apoptotic proteins such as BCL-2 [115]. These studies suggest that CS increases oxidative stress and dysregulates apoptosis in the lung, leading to the development of lung diseases such as COPD and lung cancer. Overall, smoking can dysregulate apoptosis signaling pathways, initiating lung cell apoptosis and contributing to the development of lung diseases. Understanding how smoking affects apoptosis is essential for developing effective strategies.

### 2.3. Effects of Cigarette Smoke on Cellular Senescence in the Lungs

Cigarette smoke (CS) plays a significant role in accelerating lung senescence and increasing the risk of respiratory diseases in the elderly [116]. CS contains a myriad of toxic chemicals that can induce oxidative stress, inflammation, and DNA damage, which contribute to the aging process [117].

One of the primary factors linking cigarette smoke to lung senescence is the production of reactive oxygen species (ROS) [118]. ROS is generated by the metabolism of toxic components in CS, subsequently leading to oxidative stress in lung cells [119]. Elevated ROS levels can damage cellular components such as proteins, lipids, and DNA, eventually resulting in cellular dysfunction and death [120]. In addition to oxidative stress, CS promotes inflammation in the lungs by increasing the levels of pro-inflammatory cytokines such as tumor necrosis factor and interleukin [121]. This chronic inflammation negatively impacts lung function and contributes to the development of respiratory diseases like COPD and interstitial lung disease (ILD) [122]. Cigarette smoke exposure can disrupt the balance of immune cells in the lungs, leading to chronic inflammation and impaired immune responses [123]. 

Cigarette smoke exposure can cause oxidative stress and inflammation in alveolar cells, leading to impaired surfactant production and reduced regenerative capacity [124]. Neutrophils, when activated by CS, can produce excessive ROS and release destructive enzymes, such as matrix metalloproteinases (MMPs), further contributing to lung tissue destruction and the progression of lung disease [72]. Cigarette smoke has also been found to affect key genes involved in lung senescence, such as fibronectin, MMPs, and sirtuin-1 (SIRT1) [125]. CS exposure leads to decreased fibronectin levels and increased MMP levels, which contribute to the loss of lung function and the development of respiratory diseases [126]. Furthermore, CS exposure results in the downregulation of SIRT1 in lung tissue, disrupting its protective role against lung injury and senescence [127].

Current therapeutic approaches for CS-induced lung diseases focus on managing symptoms, reducing inflammation, and improving lung function [128]. Inhaled corticosteroids, bronchodilators, and supplemental oxygen may be used for patients with COPD or ILD [129]. Enhancing the regenerative capacity of alveolar type II cells or modulating immune cell responses to reduce inflammation and oxidative stress could be potential strategies for treating CS-induced lung diseases [130]. Importantly, smoking cessation is the most effective way to prevent further lung damage and slow the progression of lung senescence [131].

Senescence and immunosenescence are interconnected concepts that all relate to aging and cellular function [132]. Cellular senescence refers to a state of permanent cell cycle arrest, which is a natural biological response to various types of stress, such as DNA damage, telomere shortening, or oncogenic stress [133]. While this process has a protective role against cancer, over time it can contribute to aging and age-related diseases [134]. Senescent cells exhibit a specific phenotype characterized by changes in morphology, gene expression, and secretion of pro-inflammatory factors, known as the senescence-associated secretory phenotype (SASP) [135]. SASP can influence the surrounding tissue microenvironment and contribute to inflammation and tissue dysfunction [136]. Senolytics are a class of drugs designed to selectively eliminate senescent cells by specifically targeting the survival pathways that these cells rely on [137]. By removing senescent cells, senolytics can alleviate SASP-induced inflammation and tissue dysfunction, potentially ameliorating the symptoms of aging and age-related diseases [132]. Preclinical studies have shown promising results, with senolytics improving health span in animal models of aging and age-related diseases, but their efficacy and safety in humans are still under investigation [138]. Immunosenescence refers to the gradual deterioration of the immune system with age, characterized by a decline in the function of the immune cells and an increase in systemic inflammation [139]. It is associated with increased susceptibility to infections, decreased response to vaccination, and a higher risk of autoimmunity and cancer in the elderly [140]. Senescent cells, through SASP, contribute to immunosenescence by creating a pro-inflammatory environment [141]. Interestingly, senescent immune cells themselves can also contribute to immunosenescence [142].

In conclusion, understanding the relationship between cigarette smoke, ROS, lung senescence, and the roles of key genes such as SIRT1 and MMPs is crucial for developing effective strategies to prevent and treat age-related lung diseases. Targeting the underlying mechanisms of senescence, including oxidative stress, inflammation, and the regulation of SIRT1 and MMPs, may lead to improved outcomes for elderly patients with respiratory diseases exacerbated by cigarette smoke exposure.

### 2.4. Understanding the Link between Cigarette Smoke and Autophagy

Autophagy is a spontaneous mechanism of decomposition that eliminates unnecessary or dysfunctional cellular components [143]. This process is crucial for maintaining cellular homeostasis through the orderly decomposition and recycling of intracellular elements [144,145]. Defects in autophagy have been linked to cancer and diabetes, with autophagy control in these diseases being studied as a potential treatment [146,147]. As autophagy is also considered an antioxidant mechanism for adapting to oxidative stress, the relationship between CS and autophagy is of particular interest [148,149]. Autophagy largely consists of five steps: initiation, elongation, autophagosome, autophagosome-lysosome fusion, and autolysosome formation [150,151]. Autophagy flux refers to the process from the autophagosome-lysosome fusion step, where degradation begins [152]. Recently, studies on the relationship between disease and disrupted autophagy flux have been actively conducted [153,154,155].

CS-induced autophagy mechanisms remain largely unknown. Recently, Wang et al. identified a link between oxidative stress caused by CS and autophagy in the human airway epithelium. They confirmed that the expression of oxidative stress-induced growth inhibitor (OSGIN1), an oxidative stress-induced growth inhibitor, was significantly increased in airway epithelial inflammation by smoking and was associated with activation of autophagy [156]. In addition, it has been reported that exposure of CS to the human alveolar epithelial cell line A549 accumulates LC3 and activates autophagy [157].

Conversely, there are reports that CS exposure impairs autophagy flux. Expression of Galectin-8, involved in initiating autophagosome engagement, increases due to activation of autophagy by CSE in macrophages. However, the accumulation of galectin-8 caused damage to autophagy flux [158]. However, the accumulation of Galectin-8 impairs autophagy flux. This accumulation is known to affect the immune response and induce cytokines and chemokines [159,160,161]. In addition, Monick et al. demonstrated that CSE treatment in human alveolar macrophages increased autophagosome production but impaired the binding process of autophagosomes and lysosomes through p62 accumulation [162]. 

As stated in numerous reports, autophagy is activates the production of autophagosomes to counteract the oxidative stress caused by CS. However, various factors can suppress the combination of the autophagosome and lysosome, which are crucial for degradation processes. For example, unlike normal oxidative stress, cadmium in cigarettes can cause the accumulation of autophagosomes [163]. This accumulation of autophagosomes results in cytotoxicity and ROS production [164]. In addition, mitophagy, which regulates the production of ROS in oxidative stress environments, is also associated with CS-induced damage [165]. Mitophagy is autophagy that occurs in mitochondria, and mitochondria are the main source of ROS, so mitophagy in oxidative stress environments is inevitable [166]. In alveolar type 2 cells, mitophagy is important for inhibiting CS-induced mitoROS that can be controlled by the expression of SIRT1 [167]. Therefore, further study of autophagy flux and mitophagy-related genes as potential treatment targets to mitigate damage to autophagy flux and mitophagy caused by CS is necessary.

## 3. CS-Induced Lung and Other Diseases

### 3.1. Chronic Obstructive Pulmonary Disease (COPD)

Chronic Obstructive Pulmonary Disease (COPD) is characterized by emphysema and chronic bronchitis [168]. Smoking is the most common cause of COPD, while other factors, such as exposure to cadmium or occupational smoke, are known to be contributors [169,170]. In addition, inorganic dust such as aluminum silicate or kaolinite in cigarettes can accumulate in alveolar macrophages and cause inflammation and pneumoconiosis [171]. Although COPD can be prevented by smoking cessation and reducing exposure to risk factors, it is not completely curable, and existing medications primarily alleviate symptoms [172].

Emphysema is a type of COPD that reduces oxygen supply to the blood by inhibiting gas exchange due to the destruction of the alveolar wall [173]. The involvement of MMPs in the destruction of alveolar walls is well known [174]. However, opinions are divided on which MMPs have a significant impact on COPD development. There are significant differences between human patients suffering from COPD and animal emphysema models [174,175]. For example, in mouse models, MMP-12 activates CXCL-5 to induce neutrophil infiltration, contributing to emphysema, but in humans, MMP12 performs the function of inhibiting the CXCL family [176]. MMP-2 has also been observed to have increased expression in the lungs and sputum of COPD patients, as well as in CS-exposed mice [177,178,179]. However, John V reported lowered MMP-2 gene expression in human lung tissue with the Global Initiative for Obstructive Lung Disease (GOLD) stage [180]. More studies are needed to resolve these discrepancies in results on the expression of MMPs and their mechanisms in COPD.

Bronchitis, a chronic mucous-producing disease in the airways, is one of the other aspects of COPD [181]. Smoking is the main cause of bronchitis, and the prevalence rate increases with years of smoking [182]. Excessive mucus resulting from inflammation in the airways can obstruct the airways. Mucus production-related genes MUC5B and MUC5AC are increasing representative factors in patients with chronic bronchitis [183,184,185]. It is difficult to conduct research on bronchitis despite the discovery of these target genes. Research on bronchitis is conducted using rat, canine, and monkey models, as the CS-induced bronchitis mouse model is nonexistent [186,187,188,189]. Therefore, the development and idea of a new disease modeling system for preclinical research on bronchitis are required.

Nuclear factor erythroid 2-related factor (Nrf2) and Kelch-like ECH-associated protein 1 (Keap1) regulating Nrf2 are regulatory redox proteins in cells [190,191]. Nrf2 and Keap1 separate in an oxidative stress environment, thereby activating Nrf2. The activated Nrf2 then translocates to the nucleus and enables expression of the Nrf2 target genes such as NQO1, heme oxygenase 1(HO1), and glutathione S-transferase (GST), which are able to eliminate ROS after binding to the promoter region of the antioxidant response elements (ARE) [192,193,194]. Since CS-induced oxidative stress and inflammation are the primary causes of COPD, Nrf2 is closely related to COPD. The importance of Nrf2 has been demonstrated in patients as well as in mouse models. It has been reported that exposure of Nrf2 knockout mice to CS made them more susceptible to emphysema [195]. Additionally, Kubo et al. reported that Nrf2 expression was observed to be lower in CS-induced emphysema mice, and treatment with astaxanthin restored the Nrf2 levels, alleviating emphysema [196]. Pasini et al. observed elevated expression of Nrf2 and HO-1 in the blood of COPD patients, accompanied by lower forced expiratory volume (FEV1) in the first second [197]. Furthermore, the genes NQO1, HO1, superoxide dismutase type 1 (SOD1), and thioredoxin reductase 1 (TXNRD1) were downregulated in COPD patients, with increased expression following treatment with Nrf2 activators [198]. These findings indicate that Nrf2 inhibition in patients or mice can result in an exacerbation of emphysema and inflammation due to an inadequate response to oxidative stress.

COPD can also be caused by the activation of various DAMPs and the subsequent inflammatory responses. Several studies have revealed that expression levels of AGEs and RAGEs are high in COPD patients. Smith et al. reported that sRAGE levels were lower in COPD patients in the presence of a negative association between FEV1 and sRAGE in a multiple linear regression analysis [199]. Hoonhorst et al. measured AGE and RAGE levels in young (18–40) as well as old (40–75) smokers, non-smokers, and COPD patients and found that the lowest sRAGE expression was seen in the plasma of COPD patients [200]. On the other hand, mRAGE is generally upregulated in COPD. Ferhani et al. reported significantly higher levels of high mobility group box 1 (HMGB1), a representative RAGE ligand, in COPD patients, with mRAGE overexpressed in airway epithelium and smooth muscle [201]. In addition, it has been reported that exposure to CS extract induces alveolar epithelial cell injury and results in an upregulation of mRAGE [42,202]. Furthermore, RAGE-/- mice exposed to cigarette smoke showed less emphysema compared to WT [203]. The differential expression of mRAGE and sRAGE in COPD patients or smokers may be genetic. In 2016, Miller et al. confirmed through single nucleotide polymorphism (SNP) analysis that a lower expression of sRAGE was observed in the UK smoker cohort, with individuals having the T allele of rs2070600, which is associated with pulmonary function [204,205,206]. 

RAGE-DAMP signaling is the initial point for various inflammatory and oxidative stress mechanisms, which then activate MAPK. It has been reported that exposure to CS results in increased inflammation, which is accompanied by increased activity of JNK and p38 in human bronchial epithelial cells [207]. Marumo et al. confirmed that CS exposure resulted in an increased mRNA expression of p38 in C57BL/6 mice, while no such effect was observed in the NZW mice (emphysema-resistant) [208]. Renda confirmed an increased expression of phosphorylated p38 in the alveoli of COPD patients [209]. Additionally, ERK expression in COPD patients induces endothelial cell apoptosis by upregulating MMP-1 and MUC5AC, leading to destruction of the alveolar wall and inflammation [210,211,212]. Taken together, RAGE-DAMP signaling can cause extensive alveolar damage and inflammation via MAPK. Therefore, inhibition of mRAGE or upregulation of sRAGE can serve as a potential target for preventing COPD.

The expression of NOX is closely related to COPD. It was confirmed that the protein expression of the NOX family in the tissues of COPD patients was significantly higher as compared to the non-smoker control group [213]. Xinjing et al. reported that NOX 1, 4, and 5 were detected at various sites such as lung epithelial cells, vascular endothelial cells, and macrophages, and NOX2 was mainly detected in lung macrophages and neutrophils [214]. Stanley et al. also reported that the NOX inhibitor apocynin inhibited lung inflammation and vascular injury caused by oxidative stress in the CS-induced mouse model [215]. In addition, expression of NOX in COPD has been reported in several studies [216,217,218]. Therefore, since NOX is a major source of ROS, targeting NOX to suppress overproduction of ROS might be an effective way to protect against oxidative stress. Dual oxidase (DUOX), a NOX homolog, is also important in COPD. Caspar et al. reported that DUOX1 is downregulated in airway epithelial cells of COPD patients and that a deficiency of DUOX1 in mice enhances emphysema [219]. In addition, Katsura Nagai et al. reported that DUOX1 was downregulated in airway epithelial cells of smokers compared to non-smokers and that both DUOX1 and DUOX2 were downregulated in bronchial epithelial cells of COPD patients [220]. Interestingly, DUOX is downregulated in COPD patients as opposed to NOX, which may be due to DUOX1 inhibiting epithelial damage and contributing to maintaining epithelial integrity [221]. In addition, in previous studies, DUOX1 is involved in epithelial damage response by MMP-9, and overexpression of MMP-9 can cause protease/antiprotease imbalance and lead to COPD [222,223]. Therefore, among NOX families, the functions of NOX series and DUOX series in COPD conflict with each other, so targeting them should be careful in the study.

Metformin and astaxanthin are among the several treatments that have recently been studied to treat COPD. Metformin, originally a treatment for type 2 diabetes, has been reported not only to increase insulin sensitivity but also to suppress damage by controlling cell redox homeostasis [224,225]. Recently, Francesca et al. reported that metformin inhibits oxidative stress and apoptosis through regulation of adenosine monophosphate (AMP) kinase signaling in CS-induced emphysema mouse models [226].

Astaxanthin is a keto-carotenoid derived from *Hematococcus pluvialis*, which is used as a health binder with Sirtuin1 (SIRT1) supplements for improving muscle strength and also has therapeutic effects on atherosclerosis and macular degeneration [227,228,229,230]. Mingming et al. reported that Astaxanthin binds with SIRT1, inhibits Nrf2-modulated oxidative stress, and regulates NF-κB-related inflammatory responses in CS-induced emphysema mouse models and human bronchial epithelial cells [231]. 

Also, new therapeutic agents or transporter studies have been reported to treat COPD. For example, Noridzada et al. treated the human umbilical cord mesenchymal system cell (HUC-MSC)-derived extracellular vesicle, which resulted in the alleviation of airway inflammation in the CS-induced rat model [232]. In addition, Emanuela et al. used lipid-polymer hybrid nanoparticles (LPHNPs) to develop a drug delivery system for Roflumilast, a representative PDE4 inhibitor [233]. In addition, studies have been conducted using liposomes and nanoparticles targeting oxidative stress for treating COPD [234,235,236,237]. These therapeutic studies will be the steppingstones for obtaining treatment for COPD.

### 3.2. Pulmonary Fibrosis

Pulmonary fibrosis is a chronic lung disease characterized by excessive deposition of extracellular matrix and progressive scarring of lung tissue. Smoking is a major risk factor for pulmonary fibrosis. The genetic and molecular mechanisms underlying CS-induced pulmonary fibrosis involve oxidative stress, inflammation, fibroblast activation, and epithelial-mesenchymal transition (EMT) [238]. Genetic polymorphisms in SNPs related to ROS metabolism, such as cytochrome P450 Family 1 subfamily A Member 1 (CYP1A1) and cytochrome P450 Family 1 subfamily B Member 1 (CYP1B1), as well as antioxidant enzymes, such as GST and SOD, increase susceptibility to CS-induced pulmonary fibrosis [239,240]. Several studies have reported the overexpression of NADPH oxidases, particularly NOX4 [241], which is believed to contribute to the development and progression of the disease [242]. Specifically, NOX4-derived ROS has been implicated in fibroblast activation and myofibroblast transformation, which are the key processes in the pathogenesis of IPF [243]. Therefore, targeting NOX4 might offer therapeutic benefits in IPF [244].

Rage is one of the several molecular players implicated in the pathogenesis of PF [245]. RAGE contributes to the maintenance of alveolar structure and function in healthy lungs. However, the overall expression of RAGE is reported to be decreased in the lung tissues of patients with PF [246]. More specifically, RAGE expression has been observed to be significantly reduced in fibrotic areas of the lungs affected by PF [247]. This lowering of RAGE expression is associated with the loss of type I alveolar epithelial cells (AECs), which are replaced by type II AECs and fibroblasts during disease progression [248]. Additionally, levels of sRAGE in the serum of PF patients are often elevated [249], which might reflect increased cleavage and loss of membrane-bound RAGE from the injured alveolar epithelium in PF [250]. Further studies are needed to fully understand the role of RAGE in PF and its potential as a therapeutic target. Overexpression of RAGE leads to an exaggerated inflammatory response, including the release of pro-inflammatory cytokines [251]. In cigarette smoke exposure, it could exacerbate the inflammatory and oxidative stress responses, thereby contributing to the progression of lung disease [252]. In IPF, overexpression of RAGE could potentially worsen fibrotic responses, as RAGE signaling has been implicated in fibroblast activation and collagen production [247]. RAGE knock-out mice often show less severe disease phenotypes in response to harmful stimuli such as cigarette smoke [253]. The knock-out mice show less inflammation and oxidative stress in response to cigarette smoke and less fibrosis in models of IPF [254]. Nuclear RAGE interacts with specific DNA repair proteins, potentially regulating their activity [255]. As a result, it may influence the cellular response to DNA damage [42].

An increased risk of pulmonary fibrosis in smokers is associated with genetic variations in genes encoding inflammatory mediators such as IL-1, TNF-α, and TGF-β [256]. Additionally, polymorphisms in the genes encoding chemokine (C-C motif) ligand 18 (CCL18) and chemokine (C-X-C motif) receptor 3 (CXCR3) have been linked to CS-induced pulmonary fibrosis [257]. Fibroblast activation causes excessive deposition of extracellular matrix components, such as collagen and fibronectin. Activation of lung fibroblasts and extracellular matrix production, such as MMPs and tissue inhibitors of metalloproteinases (TIMPs), is also a result of CS exposure [258,259]. EMT, characterized by the transformation of epithelial cells into mesenchymal cells, is a critical process in pulmonary fibrosis, resulting in increased fibroblast activity and extracellular matrix deposition. Smoking upregulates TGF-β signaling, thereby inducing EMT in lung epithelial cells [260]. Susceptibility to CS-induced pulmonary fibrosis might be influenced by genetic variations in EMT-related genes, such as Snail Family Transcriptional Repressor 1 (SNAI1), Twist Family BHLH Transcription Factor 1 (TWIST1), and Zinc Finger E-Box Binding Homeobox 1 (ZEB1).

Antioxidant therapies, such as N-acetylcysteine, have shown potential for ameliorating oxidative stress in pulmonary fibrosis [261]. N-acetylcysteine replenishes the levels of the antioxidant glutathione and reduces reactive oxygen species, thereby mitigating the harmful effects of oxidative stress on lung tissue [262]. Anti-inflammatory agents targeting pro-inflammatory cytokines, such as TNF-α, IL-1, and TGF-β [263], and anti-fibrotic drugs, including pirfenidone and nintedanib [264,265] have been explored as potential treatments for pulmonary fibrosis in addition to antioxidant therapies.

Targeting EMT with TGF-β signaling inhibitors, such as galunisertib, has shown promising results in preclinical studies [266]. Modulating specific microRNAs (miRNAs) is emerging as a therapeutic strategy for CS-induced pulmonary fibrosis and EMT [267]. It would be therapeutically beneficial to target chemokine signaling pathways such as CCL2/CCR2 and CCL18/CCR8 [268]. 

Phosphodiesterase 4B (PDE4B) inhibitors can play a significant role in alleviating disease progression and function as a therapeutic target for PF. The inflammation and fibrotic processes, which are often exacerbated by CS exposure in normal human bronchial epithelial cells, are reduced by the PDE4B inhibitors [269]. With regards to CS-induced lung damage, inhibitors targeting PDE4B might help mitigate the harmful effects of smoke and contribute to the overall treatment of pulmonary fibrosis [270].

In conclusion, CS-induced pulmonary fibrosis is a complex disease with multiple genetic and molecular mechanisms. Currently, emerging therapeutic strategies targeting EMT, miRNAs, and chemokine signaling might provide more effective treatments for this debilitating lung disease.

### 3.3. Cancers

Lung cancer is the leading cause of cancer-related deaths worldwide, accounting for approximately 1.8 million deaths per year [271]. The primary cause of lung cancer is cigarette smoking, which accounts for approximately 80–90% of all cases [272] and 25% of all cancer deaths. Smokers are 15–30 times more likely to develop lung cancer than non-smokers, and the risk increases proportionally with the number of cigarettes smoked per day and the duration of smoking [273]. Additionally, antioxidants such as glutathione, which are crucial in protecting against oxidative stress, can be depleted by CS [274]. The imbalance between ROS and antioxidants can result in chronic oxidative stress, which has been linked to lung cancer development [14]. 

Cigarette smoking, which contains more than 70 carcinogens, including polycyclic aromatic hydrocarbons and nitrosamines, has been linked to an enhanced risk of various types of cancer. Studies have indicated an association between smoking and an increased risk of bladder cancer, pancreatic cancer, kidney cancer, esophageal cancer, and head and neck cancers [272,275,276,277,278,279,280]. Oxidative stress has also been implicated in the development of many types of cancer, including lung cancer, breast cancer, and prostate cancer [281,282,283,284], and is also involved in inducing epigenetic changes, such as DNA methylation and histone modifications, which can alter expression patterns of genes and contribute to tumorigenesis [285].

In cancer, the role of NADPH oxidases is complex and depends on the type of cancer [286]. Some cancers are associated with enhanced expression of certain NOX isoforms, which contribute to cancer progression by promoting cell proliferation, survival, angiogenesis, and metastasis [287]. For example, NOX1 is upregulated in colon cancer, while NOX4 and NOX5 are often overexpressed in breast cancer [288]. Furthermore, the reactive oxygen species produced by these enzymes can result in DNA damage, which can further lead to mutations and the development of cancer [289].

NF-κB is a key factor related to controlling inflammation caused by oxidative stress, which was caused by CS [290]. CS reportedly increases the expression of inducible nitric oxide synthase (iNOS) and activates NF-κB, causing inflammation in human lymphocytes [291]. Oxidative stress-induced activation of the NF-κB pathway is involved in tumorigenesis by enhancing c-Myc and cyclin D1 levels [292,293]. The DNA damage induced by ROS results in mutations and altered gene expression patterns that promote cell proliferation and survival, including the activation of oncogenes and the inactivation of tumor suppressor genes [294]. The hypoxia-inducible factor (HIF) pathway, which plays a critical role in tumor angiogenesis, metastasis, and resistance to chemotherapy, can also be activated by ROS [295]. The PI3K/AKT/mTOR pathway, which regulates cell survival and proliferation [296], and the dysregulation of which has been implicated in several cancers, including lung cancer, is activated by ROS [297]. The redox state imbalance is characteristic of cancer, and the role of the antioxidant factor Nrf2 is important [298]. Interestingly, Nrf2 plays a dual role in cancer. In general, in the early stages of cancer, NRF2 activates DNA damage, cell cycle arrest, and DNA repair [299,300], and with the progression of the tumor, the activity of NRF2 can contribute to protecting against oxidative stress in tumor cells [300,301]. Therefore, treatment targeting Nrf2 can be either effective or suppressive, depending on the progression of the cancer.

Animal models have been developed to determine key molecular pathways involved in lung cancer, such as the PI3K/AKT/mTOR pathway and hypoxia-inducible factors, and test the efficacy of various therapies. Nano-immunotherapy has been observed to enhance the antitumor immune response and enhance therapeutic outcome in preclinical lung cancer models [302,303]. Zhong et al. reported that apoptosis, along with inhibition of NF-κB, accumulation of IκBα, and reduction in the DNA binding activity of NF-κB, was observed in the lungs of cigarette-exposed mice [304]. Clinical trials demonstrated that targeted therapy with epidermal growth factor receptor (EGFR) inhibitors improved progression-free survival and overall survival in patients with non-small cell lung cancer with EGFR mutations [305]. Moreover, some patients achieved long-term remission when treated with immunotherapy with checkpoint inhibitors, such as pembrolizumab and nivolumab, in clinical trials [306,307].

The aim of cancer treatment strategies is to improve upon traditional therapies like dexamethasone, a steroid that is often used to reduce inflammation and suppress the immune response in various cancers [308]. PD-1/PD-L1 inhibitors act by potentially mitigating oxidative stress, thereby improving the therapeutic efficacy of traditional therapies. Reduced oxidative stress for PD-1/PD-L1 inhibitors may enhance the ability of the immune system to target cancer cells by preventing immunosuppressive effects in the tumor microenvironment. This can be achieved by combining antioxidant therapies with PD-1/PD-L1 inhibitors to lower ROS levels and restore immune cell functionality [309]. The advent of novel therapeutic modalities such as nanocarriers, antibodies, gene editing technologies, and exosome technologies has led to a significant evolution of the cancer treatment landscape. Liposomes are a type of nanocarrier that is widely used to safely transport drugs and deliver them to specific tissues [310]. Monoclonal antibodies (mAbs) [311], which can function by directly inhibiting cancer cell growth, inducing apoptosis, or stimulating the immune system to attack the cancer cells, have emerged as one of the most successful strategies for targeted cancer therapy [312]. Examples of monoclonal antibodies include trastuzumab for HER2-positive lung cancer and rituximab for B-cell malignancies [313,314]. Gene editing, particularly the CRISPR-Cas9 system, is a powerful tool in cancer research and therapy [315]. This technology can be used not only to modify or correct disease-causing mutations in cancer cells [316], but it can also be used to engineer immune cells, such as T cells, to enhance their ability to recognize and kill cancer cells—a strategy used in CAR-T cell therapy [317]. Combining CAR-T-cell therapy with antioxidant treatments enables the management of oxidative stress by neutralizing ROS and enhancing the immune response of tumors, thereby improving the survival and functionality of engineered T cells [318]. Exosomes are small vesicles secreted by cells that can carry proteins, lipids, and nucleic acids [319], which can be engineered to deliver therapeutic agents, such as drugs or RNA molecules, directly to cancer cells [320]. Exosomes derived from mesenchymal stem cells (MSCs) exert therapeutic effects in the lungs [321]. These novel therapeutic strategies, though promising, also face challenges such as potential side effects, difficulty in delivery, and issues with specificity and efficiency. Ongoing research is needed to address these challenges and enable the full realization of the potential of these technologies in cancer treatment. 

### 3.4. Acute Respiratory Distress Syndrome and Acute Exacerbations of COPD

Acute respiratory distress syndrome (ARDS) is a type of respiratory failure characterized by extensive lung inflammation that is caused by sepsis, trauma, pneumonia, and aspiration damage [322]. Pathophysiology involves an inflammatory cascade and destruction of the air-blood barrier [323]. During this process, the infiltrated neutrophils produce neutrophil elastase (NE) and MMPs and damage the air-blood barrier, leading to a vicious cycle leading to more inflammation and edema [324,325,326]. 

Although a direct association between smoking and ARDS is not known, studies suggest that CS exposure in ARDS patients increases the risk and severity. Moazed et al. reported that patients with ARDS exposed to CS had elevated plasma IL-8 levels [327,328]. In addition, exposure of smokers or non-smokers to LPS resulted in elevated levels of IL-1β, IL-8, and more excess neutrophils in the BAL of smokers [329]. Liu et al. observed an increased expression of angiotensin-converting enzyme 2 (ACE2), the receptor for SARS-CoV-2, in human airway epithelial cells [330,331]. A meta-analysis conducted by Cai et al. confirmed higher ACE2 gene expression in the lungs of smokers than in non-smokers [332]. Voinsky et al. demonstrated an enhanced expression of transmembrane proteases serine 2 and 4 (TMPRSS2 and TMPRSS4), which are important for SARS-CoV-2 to enter the cell, as well as ACE2 in the bronchi of smokers [333]. Their findings suggest that smoking may increase the risk of severe COVID-19 [334].

Recently, it has been reported that vitamin C has a positive effect on ARDS. Intravenous injections of vitamin C regulate neutrophil extracellular traps (NETs) in ARDS patients, thereby attenuating the ARDS-related biomarker synndecan-1 [335]. Vitamin C is one of the leading antioxidants and is important for the functioning of the immune system [336]. Activation of NADPH oxidase via ROS causes NETs [337]. Furthermore, various studies have reported administering ARDS treatment by using nanocarrier drug delivery systems. Saiping et al. developed a nanostructured lipid carrier that combines the intercellular adhesion molecule 1 (ICAM-1) antibody (ICAM/NLC) and confirms low pro-inflammatory cytokine levels in the ARDS-mouse model [338]. The development of such treatment modalities is important for overcoming ARDS.

Sudden deterioration of respiratory function in COPD patients is referred to as acute exacerbations of chronic obstructive pulmonary disease (AE-COPD) [339]. This exacerbation worsens respiratory failure, necessitating mechanical ventilation [340]. Bacterial infections are commonly associated with AE-COPD [341,342,343,344] and COPD patients due to their compromised airways. Wang et al. categorized AE-COPD patients as smokers or non-smokers and conducted an analysis of the hematological parameters, which demonstrated that smokers exhibited higher counts of eosinophils and basophils in BAL [345]. Moreover, Li and colleagues reported that, as compared to smokers, non-smokers with AE-COPD had higher FEV1/forced vital capacity (FVC) and experienced less wheezing and phlegm production [346]. Thus, it is evident that CS-induced lung damage negatively impacts both chronic and acute inflammatory lung diseases, both before and after their onset.

### 3.5. Cardiovascular Disease

Cardiovascular disease (CVD) encompasses various heart and vascular conditions, such as angina, myocardial infarction, coronary artery disease, and heart failure [347]. Numerous reports have reported CS-induced adverse effects on cardiovascular disease [348,349,350,351,352]. However, the specific mechanisms that exist between CS and cardiovascular disease have not been fully elucidated.

CS exposure increases the risk of atherosclerosis and, consequently, coronary artery syndrome and stroke, as it causes vascular dysfunction and inflammation. Several reports have shown that CS exposure impairs endothelial-dependent vasodilation in humans [353,354]. It has also been found that CS reduces the synthesis of nitrogen oxide (NO) [355,356]. In general, NO, along with guanosine monophosphate (GMP) and calcium channels, is involved in vasodilation [357]. In vitro studies have demonstrated lower endothelial NOS (eNOS) activity and decreased production of NO when endothelial cells of the human coronary artery were treated with the serum of smokers [358]. Furthermore, CS-induced ROS activates the NOD-like receptor (NLR) family pyrin domain containing 3 (NLRP3), inducing the expression of IL-1β and IL-18, resulting in autophagy, apoptosis, and endothelial cell dysfunction [359,360]. In addition, CS can secrete several cytokines, thereby affecting white blood cell recruitment. Mazzone reported that elevated levels of soluble intercellular adhesion molecule 1 (sICAM-1) and soluble vascular cell adhesion molecule 1 (sVCAM-1), which could contribute to hypertension, were identified in the plasma of smokers [361]. Smoking can also increase low-density lipoprotein (LDL) levels in the blood, leading to the formation of oxidized LDLs (oxLDL), which in turn leads to the production of pro-inflammatory cytokines and foam cells [362,363,364]. Foam cells are generated when oxLDL functions as a ligand in the lectin-like oxidized LDL receptor-1 (LOX-1), which is a macrophage-expressing receptor. Smoking increases the expression of LOX-1 [365,366]. The accumulation of foam cells can lead to atherosclerosis [367]. 

## 4. Conclusions

**Figure 1:**
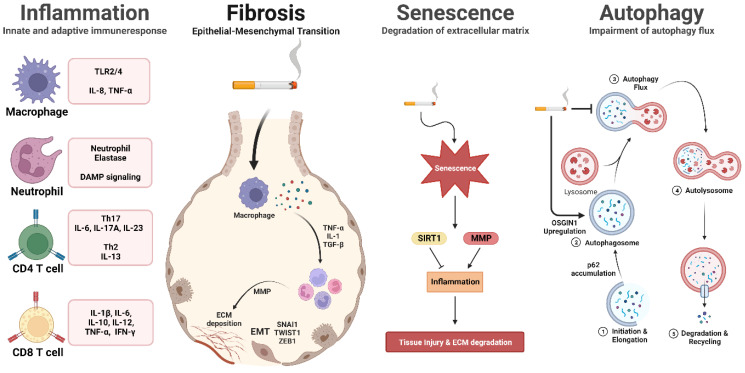
Cigarette smoke-mediated cellular pathways in inflammation, fibrosis, senescence, and autophagy. Cigarette smoke exposure initiates inflammation pathways, leading to the recruitment of immune cells and the subsequent production of pro-inflammatory cytokines. Prolonged exposure to cigarette smoke activates fibrosis pathways, resulting in extracellular matrix deposition and fibrosis formation. Moreover, cellular senescence is expedited, and the autophagy pathway is modified in response to cigarette smoke, contributing to the onset of age-related diseases. This illustration depicts the cellular pathways influenced by cigarette smoke exposure.

In this review, we explored the cigarette smoke-mediated cellular pathways, focusing on NF-κB, MAPK, Nrf2, and RAGE. Furthermore, HIF, mTOR, TGF-β, and NLRP3 play key roles in respiratory diseases such as cancer, pulmonary fibrosis, and cardiovascular diseases. These factors are influenced by oxidative stress within the body, resulting in a variety of harmful effects. These complex and pathogenic cellular processes negatively impact various diseases, including multiple types of cancer, chronic and acute lung diseases, and cardiovascular diseases. Recently, focus has shifted beyond traditional methods, and novel treatment modalities such as nanocarriers, drug delivery systems, mAbs, gene editing, and exosomes have gained attention. We have summarized the various therapy modalities for CS-induced lung diseases in Figure 1 and Table 1. Future research targeting these damage mechanisms may pave the way for the development of therapeutic modalities to mitigate the effects of these diseases.

## Figures and Tables

**Table 1:** Treatment approaches for cigarette smoke-induced lung diseases.

Respiratory Disease	Therapy	CS-Induced Mechanism	Reference
COPD	Metformin	Inhibition of apoptosis through regulation of AMP kinase.	[226]
	Astaxanthin	Inhibition of Nrf2-modulated oxidative stress and regulation of NF-κB-related inflammatory responses through binding with SIRT1.	[231]
	HUC-MSC- derived EVs	Alleviation of airway inflammation in the CS-induced rat model.	[232]
	LPHNPs	Upregulation of cytocompatibility toward bronchial epithelial cells and macrophages.	[233]
Pulmonary Fibrosis	N-acetylcysteine	Replenish the levels of the antioxidant glutathione and reduce reactive oxygen species by targeting pro-inflammatory cytokines such as TNF-α, IL-1, and TGF-β.	[262,263]
	PDE4B inhibitors	Reducing inflammation and fibrotic processes by inhibiting the degradation of cAMP.	[269]
Cancer	PD-1/PD-L1 inhibitors	Enhance the immune system’s ability to target cancer cells and restore immune cell functionality by combining PD-L1 from cancer and PD-1 from T cells.	[309]
	Trastuzumab	Durable anticancer activity in patients with previously treated *HER2*-mutant of NSCLC.	[291]
	Rituximab	Depletes CD20-positive B cells in lung tumors.	[292]
	CRISPR-Cas9	Modify or correct disease-related genes causing mutations in cancer cells.	[316]
	CAR-T-cell	improve the survival and functionality of engineered T-cells by reducing ROS and eliminating cancer cells.	[318]
	MSCs-exosome	Cell-to-cell communication within the tumor microenvironment and suppression of angiogenesis.	[321]
ARDS and AE-COPD	Vitamin C	Antioxidant vitamin C inhibits ROS-mediated NET by inactivating NADPH oxidase.	[335]
	ICAM/NLC	Decrease pro-inflammatory cytokines in the ARDS mouse model.	[338]

## Abbreviations

**Abbreviation**	**Definitions**
CS	cigarette smoke
ROS	reactive oxygen species
COPD	chronic obstructive pulmonary disease
PF	pulmonary fibrosis
WHO	World Health Organization
FCTC	Framework Convention on Tobacco Control
NOX	NADPH oxidase
NETs	neutrophil extracellular traps
DAMP	damage-associated molecular patterns
RAGE	receptor for advanced glycation end products
HMGB1	high mobility group box 1
sRAGE	soluble form RAGE
mRAGE	membrane-bound form RAGE
TLRs	toll-like receptors
H1R	histamine receptor-1
COX2	cyclooxygenase2
MAPK	mitogen-activated protein kinase
NF-κB	nuclear factor kappa-light chain-enhancer of activated B cells
IKK	IκB kinase
ERK1/2	extracellular signal-regulated kinases
JNK1/2	c-Jun N-terminal Kinase
STAT	signal transducer and activator of transcription
IRF9	interferon regulatory factor 9
CBP	CREB-binding protein
CSE	cigarette smoke extract
BAL	bronchoalveolar lavage
BCL-2	B-cell lymphoma 2
TP53	tumor protein p53
IAPs	inhibitor of apoptosis proteins
TSSK4	testis-specific serine/threonine kinase 4
NQO1	NAD(P)H dehydrogenase [quinone] 1
AKR1C3	aldo-keto reductase family 1 member C3
GPX2	glutathione peroxidase 2
ILD	interstitial lung disease
MMPs	matrix metalloproteinases
SIRT1	sirtuin-1
SASP	senescence-associated secretory phenotype
OSGIN	oxidative stress-induced growth inhibitor
Nrf2	nuclear factor erythroid 2-related factor
Keap1	Kelch-like ECH-associated protein 1
ARE	antioxidant response elements
HO1	heme oxygenase 1
GST	glutathione S-transferase
FEV1	forced expiratory volume
SOD1	superoxide dismutase type 1
TXNRD1	thioredoxin reductase 1
GOLD	Global Initiative for Obstructive Lung Disease
HUC-MSC	Human umbilical cord mesenchymal system cell
LPHNPs	lipid-polymer hybrid nanoparticles
EMT	epithelial-mesenchymal transition
SNPs	single nucleotide polymorphisms
CYP1A1	cytochrome P450 Family 1 Subfamily A Member 1
CYP1B1	cytochrome P450 Family 1 Subfamily B Member 1
CCL18	chemokine (C-C motif) ligand 18
CXCR3	chemokine (C-X-C motif) receptor 3
TIMPs	tissue inhibitors of metalloproteinases
miRNAs	microRNAs
PDE4B	phosphodiesterase 4B
iNOS	inducible nitric oxide synthase
HIF	hypoxia-inducible factor
NSCLC	non-small-cell lung cancer
mAbs	monoclonal antibodies
MSCs	mesenchymal stem cells
PAHs	polycyclic aromatic hydrocarbons
ARDS	acute respiratory distress syndrome
NE	neutrophil elastase
ACE2	angiotensin-converting enzyme 2
TMPRSS2/4	transmembrane protease, serine 2 and 4
ICAM-1	Intercellular adhesion molecule 1
AE-COPD	acute exacerbations of chronic obstructive pulmonary disease
FVC	forced vital capacity
CVD	cardiovascular disease
GMP	guanosine monophosphate
eNOS	endothelial NOS
NLR	NOD-like receptor
NLRP3	NLR family pyrin domain containing 3
sVCAM-1	soluble vascular cell adhesion molecule 1
LDL	low-density lipoprotein
oxLDL	oxidized LDLs
LOX-1	lectin-like oxidized LDL receptor-1
